# Cohort study to evaluate efficacy, safety and acceptability of a two-rod contraceptive implant during third, fourth and fifth year of product use in China

**DOI:** 10.1016/j.conx.2019.100008

**Published:** 2019-06-20

**Authors:** Y Che, D Taylor, D Luo, LY Maldonado, M Wang, S Wevill, H Vahdat, X Han, V Halpern, L Dorflinger, MJ Steiner

**Affiliations:** a Key Laboratory of Reproduction Regulation of NPFPC (SIPPR, IRD, Fudan University), Shanghai 200032, China; b Contraceptive Technology Innovation Division, FHI 360, 359 Blackwell Street, Durham, NC 27701, USA; c Henan Provincial Research Institute for Population and Family Planning, Henan, 450002, China

**Keywords:** Sino-implant, Levoplant, Long-acting reversible contraceptive, Contraceptive effectiveness, safety

## Abstract

**Objective:**

Sino-implant (II) is a contraceptive implant approved for 4 years of use in China. We evaluated the contraceptive efficacy during the third, fourth and fifth year, and assessed additional pharmacokinetics (PK), safety, and acceptability endpoints.

**Study design:**

We enrolled a cohort of 255 current Sino-Implant (II) users entering their third year and a second cohort of 243 users entering their fourth year. We followed these two cohorts for 12 and 24 months, respectively. To characterize PK endpoints (i.e. levonorgestrel (LNG), sex hormone binding globulin and free LNG index) over 5 years, we collected blood samples in a subset of 50 participants we followed during the third, fourth and fifth year. We also enrolled small cohorts (n = 20) of Sino-implant (II) users entering their sixth month and second year and followed them each for up to 6 months. Our primary efficacy measures were the pregnancy Pearl Indices during Year 3 and 4. Secondary objectives included assessments of PK, safety, acceptability and efficacy in the fifth year.

**Results:**

We recorded four pregnancies, with a higher pregnancy rate during Year 3 [1.34 (95% CI: 0.28–3.93)] than Year 4 [0.44 (95% CI: 0.01–2.47)] or Year 5 [0.00 (95% CI: 0.00–2.02)]. The overall pregnancy rate for the third, fourth and fifth years of product use was 0.63 per 100 WY; 95% CI: (0.17–1.62). Mean LNG concentrations remained well above 200 pg/mL (Year 3 = 280.9; Year 4 = 233.6; Year 5 = 270.6). Most participants (93.7%) described their bleeding pattern as acceptable.

**Conclusion:**

Sino-implant (II) is a highly effective contraceptive method in this population of Chinese women over 5 years.

**Implications:**

Sino-implant (II) is a highly effective contraceptive method with an estimated Pearl Index of less than 1% over the third, fourth and fifth years of use in a population of Chinese women of reproductive age.

## 1 Introduction

Sino-implant (II) is a subdermal contraceptive implant manufactured in China (Shanghai Dahua Pharmaceutical Co., Ltd. (Dahua)) and now marketed globally as Levoplant™. The product consists of two flexible silicone rods loaded with 75 mg of levonorgestrel (LNG) — 150 mg LNG per set. Sino-implant (II) has been approved in China since 1997, with a labeled four-year duration of use based on data collected over 20 years ago in China [1]. Sino-implant (II) has a similar design, the same active pharmaceutical ingredient (API), and the same dose of API as Jadelle® (Bayer Healthcare, Berlin, Germany), which is approved for 5 years of use by stringent regulatory authorities, including the United States Food and Drug Administration (USFDA).

Until recently, the high price of Jadelle® prevented its wide-scale adoption in developing countries [2]. To introduce competition, we undertook steps to obtain World Health Organization (WHO) prequalification (PQ) of a lower-cost Chinese implant. WHO PQ or approval by a stringent regulatory authority is necessary for global procurement agencies (e.g., UNFPA and USAID) to distribute the product.

We have reported previously on the pivotal trial conducted in the Dominican Republic (DR), used for WHO PQ of Levoplant™ [3]. In this article, we present results from a cohort study originally designed to provide supportive data to the DR trial that enrolled existing Sino-implant (II) users in China. The primary objective of this study was to evaluate the contraceptive efficacy of Sino-implant (II) during Years 3 and 4. Secondary objectives included assessments of pharmacokinetics (PK), safety, acceptability and efficacy during the fifth year of use.

## 2 Methods

### 2.1 Study design and enrollment

We conducted this observational study in four state-run family planning clinics in China (Tongxiang, An Yang, Lingbao, Lushi). The ethical review boards at FHI 360 and the Shanghai Institute of Planned Parenthood Research (SIPPR) approved the protocol and we registered the study on ClinicalTrials.gov (NCT01936454).

We contacted in person and by telephone existing Sino-implant (II) users entering their 6th month (3–5 months post-insertion — PK Cohort 1), 2nd year (9–11 months post-insertion — PK Cohort 2), third year (21–24 months post-insertion — Year 3 Pregnancy Cohort) and fourth year (33–36 months post-insertion — Year 4–5 Pregnancy Cohort) of use from contact lists generated by the Principal Investigator (YC). These contact lists were based on chart reviews of women who had received Sino-implant (II) insertions at the study clinics as part of routine family planning services. To be eligible to be screened for the study, potential participants had to have an insertion date in their chart consistent with timeline criteria for a designated cohort and age requirements (20–44 years). We reviewed the study's purpose and procedures, eligibility criteria, and potential risks and benefits with all potential participants in person during the enrollment visit. We then consented potentially eligible women and confirmed implant placement for all enrollees with a physical exam (see Supplement for all inclusion/exclusion criteria). Participants received compensation to cover time and transportation.

### 2.2 Specimen collection

We confirmed negative pregnancy status at enrollment with a rapid urine pregnancy test (human chorionic gonadotropin (hCG) colloidal gold label manufactured by Shanghai Uppergold Bio-Pharmaceutical Co., Ltd. — QuickVue One-Step hCG Combo test). The rapid pregnancy test is highly accurate and detects hCG concentrations of 25 mIU/ml and greater (sensitivity and specificity ≫ 99%). The urine pregnancy test was repeated at the final visit of each participant exiting her cohort, and at any other visit where there were signs of pregnancy. A positive urine test was confirmed by ultrasound and/or serum quantitative hCG measurement.

We scheduled follow-up visits for the Year 3 Pregnancy Cohort at 30 and 36 months post-insertion, and at 42, 48, 54 and 60 months post-insertion for the Year 4–5 Pregnancy Cohort. PK Cohorts 1 and 2 contributed blood samples at the initial enrollment visit and at one follow-up visit 2–6 months later, such that the average sampling time was approximately 6 (PK Cohort 1) or 12 (PK Cohort 2) months post-insertion.

### 2.3 Study size determination

We targeted a sample size of 250 women in the Year 3 Pregnancy Cohort and 300 in the Year 4–5 Pregnancy Cohort to assure at least 225, 270 and 216 woman-years contributed to the Pearl Index calculation in years three, four and five of implant use, respectively. Based on these numbers, if the observed pregnancy rates during the third and fourth years of use were less than 1 per 100 woman-years, then the half-widths of the respective 95% confidence intervals would not exceed 2.3% in Year 3 or 2% in Year 4. Conservatively assuming a standard deviation of 150 throughout Year 1 based on our review of LNG data from the past Sino-implant (II) studies, enrolling 20 women in each of the PK Cohorts was expected to provide precision of about +/− 50 pg/mL when averaging each participant's two sampling time-point results. Including a subset of 20 women from the Year 3 Pregnancy Cohort and 30 women from the Year 4–5 Pregnancy Cohort also provided an expected precision of about +/− 50 pg/mL at any given sampling time point, so long as the standard deviation of total LNG levels did not exceed 125 and continuation rates were as assumed in the two pregnancy cohorts.

### 2.4 Statistical analyses of primary endpoint - pregnancy

Our primary efficacy measures were the pregnancy Pearl Indices (number of pregnancies per 100 women-years of follow-up) during the third and fourth years of Sino-implant (II) use. We censored participant time from our primary analysis at conception; at the end of the third year (Year 3 Pregnancy Cohort) or end of the fifth year of use (Year 4–5 Pregnancy Cohort) if women did not experience pregnancy post-insertion; or when they discontinued implant use or at the date of their last study visit - whichever occurred earliest. We reported the pregnancy Pearl Index with 95% confidence intervals (CI) based on a Poisson assumption for mean time to event. Secondary efficacy measures included Year 5 Pearl Index and Year 3–5 combined Pearl Index.

### 2.5 Statistical analyses of secondary endpoints - PK

The PPD bio-analytical lab measured total plasma LNG concentrations using a validated high-performance liquid chromatography tandem mass spectrometry (HPLC/MS/MS) assay (inter- and intra-assay precision, expressed as the coefficient of variation times 100, ranged from 2.72 to 6.04% and from 1.60 to 9.00%, respectively) and serum sex hormone binding (SHBG) using an ADVIA Centaur solid phase two-site chemiluminescent immunoassay. We reported the free LNG index (FLI = (LNG/SHBG*100) at each scheduled visit and summarized these data over time in graphical form.

For PK Cohorts 1 and 2, we averaged data from each participant's two sampling time points prior to analysis. Participants who discontinued or were lost to follow-up prior to contributing a second specimen had their single measurement included in the analysis if it was taken within 2 months of the target sampling time. For the 50 members recruited from the pregnancy cohorts who collectively contributed PK data, we summarized individual total LNG and SHBG measurements sampled at months 24, 30, 36, 42, 48, 54 and 60, excluding measurements taken more than 3 months from the target sampling time. We reported means, medians, minima and maxima together with two-sided 95% confidence intervals based on a log-normal distribution assumption.

### 2.6 Statistical analyses of secondary endpoints — Safety and acceptability

We asked participants about complications and known side effects of the implant during follow-up visits and collected only serious adverse event data given the product is approved in China. We assessed acceptability by calculating the proportion of study participants discontinuing Sino-implant (II), by determining primary reasons for early removal at the final visit and self-reported perceptions of bleeding pattern using questions from a recent WHO contraceptive implant trial [4].

## 3 Results

### 3.1 Study subjects

**Fig. 1:**
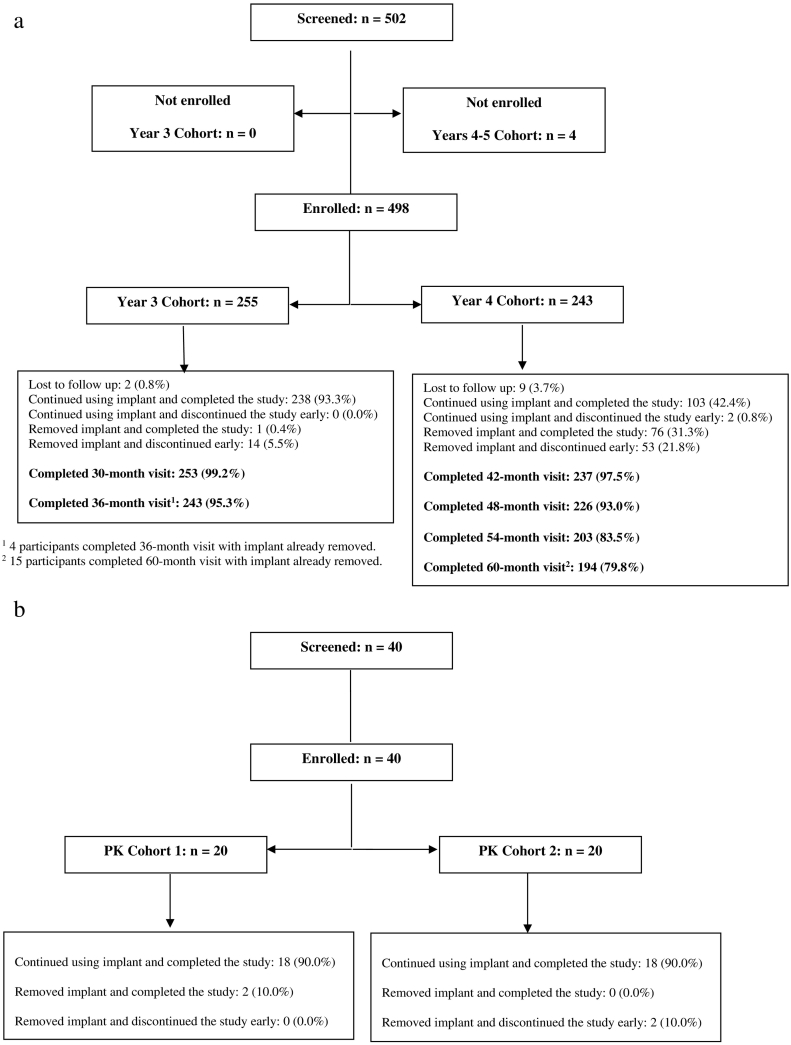
a: Participant flow diagram for a cohort study of women already using Sino-implant (II) to evaluate the contraceptive efficacy, safety and acceptability during third, fourth and fifth Year of Product Use in China (Year 3 and Years 4–5 Pregnancy Cohorts). b: Participant Flow Diagram for a cohort study of women already using Sino-implant (II) to evaluate PK outcomes at Month 6 and Month 12 (PK Cohort 1 and 2).

We screened 542 women (Fig. 1a and b) between July–October 2013 to enroll a total of 538 (99%) participants in Cohort Year 3 (n = 255), Cohort Year 4 (n = 243), PK Cohort 1 (n = 20) and PK Cohort 2 (n = 20). Four participants did not participate in the study because they lived too far from the study clinics. The follow-up was completed in November 2015. We present baseline demographic data for the two pregnancy cohorts in Table 1. Baseline demographic data for all PK groups were similar to the entire group (See Supplement for PK baseline demographic data).

**Table 1:** Baseline characteristics of women enrolled into Year 3 cohort and Year 4–5 cohort who were already using Sino-implant (II)

Variable	Year 3 cohort (n = 255)		Years 4–5 cohort (n = 243)	
Mean age, y (range)	33.9	(21–44)	36.0	(24–44)
Partner status, n (%) married or cohabitating	255	(100)	242	(99.6)
Mean body mass index kg/m^2^ (range)	23.7	(15–36)	23.7	(17–36)
Gravidity ≪ 1, n (%)	0	(0)	0	(0)
Regular menses, n (%)	234	(91.8)	223	(91.8)

### 3.2 Efficacy

We detected four pregnancies during the study (Table 2). One of three pregnancies in the Year 3 Cohort had an estimated date of conception a few days after the end of her Year 3 anniversary. Instead of censoring this endpoint per analysis plan, we classified it as a Year 3 pregnancy given the imprecise nature of pregnancy dating and our desire for a conservative approach to ensure this pregnancy was counted in our analysis. We calculated the Year 4 Pearl Index from an entirely different cohort so the pregnancy could not be added there. The fourth pregnancy we recorded in the Year 4–5 Cohort during the fourth year.

**Table 2:** Pregnancies of women enrolled into Year 3 cohort and Year 4–5 cohort who were already using Sino-implant (II)

Cohort	Date of insertion	Month to fertilization	Age	Weight	BMI	Chemical pregnancy
Year 3	09JUL11	36.3	41	75	30.0	Yes
Year 3	13JUL11	28.4	37	72	26.4	No
Year 3	29SEP11	33.7	34	54	19.6	Yes
Years 4–5	02SEP10	41.9	30	68	25.6	No

Two of the four pregnancies (both detected at the exit visit in the Year 3 Pregnancy Cohort where we tested all women regardless of symptoms of pregnancy) were chemical pregnancies that no longer were detectable by ultrasound done after the initial urine pregnancy test. In subsequent non-study clinic visits these two participants presented with no signs of pregnancy. Again, erring on the side of caution we included the two chemical pregnancies as outcomes in our primary analysis.

There were no fetal or neonatal abnormalities reported for any of the study pregnancies. The mean BMIs of the Year 3 and Year 4–5 Cohorts were in the normal range (Table 1). Of note, however, two of the four women who became pregnant were considered overweight per US Centers for Disease Control and Prevention guidelines (i.e., BMI 25–29.9) and a third woman was considered obese (i.e., BMI ≥ 30) [5].

In the primary efficacy analysis, the 248 participants in the Year 3 Pregnancy Cohort contributed 223.3 women years (WY) of follow-up and the 235 participants enrolled in the Year 4–5 Pregnancy Cohort contributed 226 WYs of follow-up in the fourth year of use, resulting in three and four-year pregnancy rates of 1.34 per 100 WY (95% CI: 0.28–3.93) and 0.44 per 100 WY (95% CI: 0.01–2.47), respectively. A total of 214 women in the Year 4–5 Cohort contributed 182.2 WYs of follow-up in the fifth year of use, resulting in a five-year pregnancy rate of 0.00 per 100 WY (95% CI: 0.00–2.02) (Table 3). This resulted in a combined 3–5 year pregnancy rate of 0.63 per 100 WY (95% CI: 0.17–1.62). In a sensitivity analysis that excluded the two chemical pregnancies, the pregnancy rate was 0.32 per 100 WY (95% CI: 0.04–1.14) over the three-year period.

**Table 3:** Pearl indices, by year of implant use and overall of women enrolled into Year 3 cohort and Year 4–5 cohort who were already using Sino-implant (II).

Time period/group	Women†	WY of follow-up	Pregnancy events	Pearl Index (per 100 WY)	95% CI for Pearl Index
**Third year of use**	248	223.3	3	1.34	(0.28, 3.93)
**Fourth year of use**	235	226.0	1	0.44	(0.01, 2.47)
**Fifth year of use**	214	182.2	0	0.00	(0.00, 2.02)
**Years 3–5, combined**	483	631.5	4	0.63	(0.17, 1.62)

† Number of women who were followed for at least one day within certain year of use and were with clear pregnancy status.

### 3.3 Pharmacokinetics

**Fig. 2:**
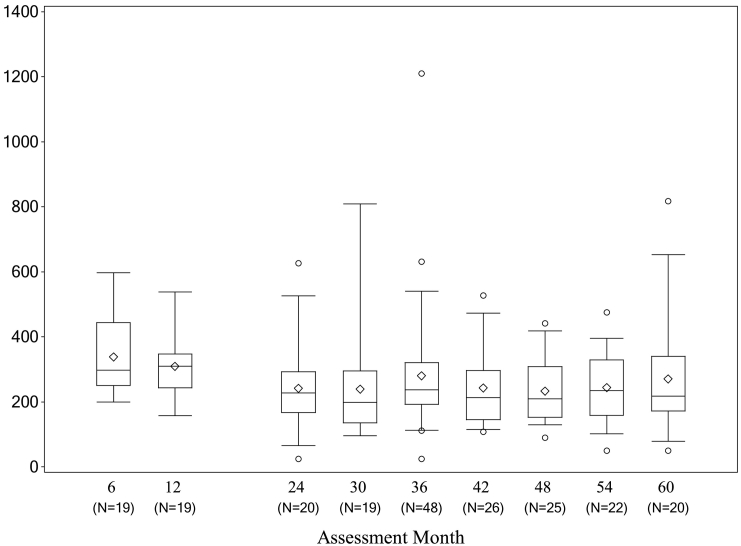
Total LNG concentrations (pg/mL) over 60 months of Sino-implant (II) use (boxes denote 25th, 50th and 75th percentiles; ◊ denotes arithmetic means; whiskers denote 5th and 95th percentiles; ○ denotes extremes).

**Fig. 3:**
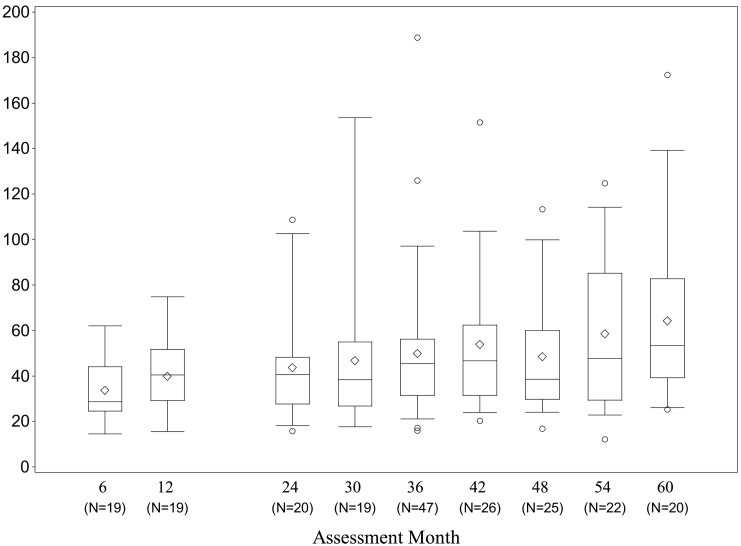
SHBG concentrations (nmol/L) over 60 months of Sino-implant (II) use (boxes denote 25th, 50th and 75th percentiles; ◊ denotes arithmetic means; whiskers denote 5th and 95th percentiles; ○ denotes extremes).

**Fig. 4:**
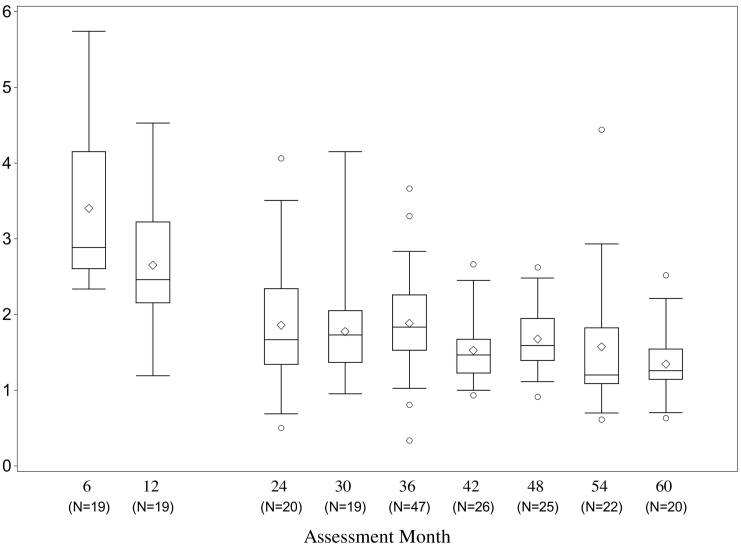
Free LNG Index over 60 months of Sino-implant (II) use (boxes denote 25th, 50th and 75th percentiles; ◊ denotes arithmetic means; whiskers denote 5th and 95th percentiles; ○ denotes extremes).

Mean total LNG concentrations decreased from 339.0 pg/mL at month 6, to 242.7 pg/mL at month 24, and remained relatively stable through month 60 (270.6 pg/mL) (Fig. 2), although there was considerable inter-subject variability (coefficients of variation exceeding 50% at most time points). In contrast, mean SHBG concentrations generally increased from 33.7 nmol/L at month 6, to 43.7 nmol/L at month 24, and 64.3 nmol/L at month 60 (Fig. 3), leading to a decrease of free LNG index values over time from a high of 3.4 at month 6, to 1.9 at month 24, and 1.3 at month 60 (Fig. 4).

### 3.4 Safety

We recorded a total of three SAEs that we determined unrelated to implant use: a sub-mucosal leiomyoma, a knee injury, and an epileptic seizure.

### 3.5 Acceptability

Most participants (91.8%) reported having had regular menses prior to using hormonal contraceptives (Table 1). When asked this same question while using Sino-implant (II) during the study, 53.8% reported having regular menses, while 41.1% said their menses were irregular (spotting only = 3.6%; no bleeding = 1.4%). Changes in bleeding patterns were generally well tolerated with most participants (93.7%) describing their bleeding pattern as “acceptable.”

Seventy-one participants (14.2%) discontinued the study early with two participants continuing to use their implants. Most common reasons for discontinuing implant use during the study included bleeding disturbances (42.0%), decreased bleeding/amenorrhea (13.0%), end of product duration (i.e., product is labeled for 4 years and participants were asked to continue using the product during the fifth year after being counseled about duration of use and providing informed consent) (23.2%), and other medical reasons (13.0%). A majority of participants (53%) who completed five years of use decided to continue using Sino-implant (II) into the sixth year.

### 3.6 Implant insertion and removal

Sixty-five participants (13.1%) complained about problems at the insertion site during the study; none led to discontinuation. The most frequent problems cited by participants included burning, prickling or numbness (n = 28; 5.2%) and sensitivity of insertion arm (n = 17; 3.2%). Clinicians reported six implants broke during 105 documented removals (5.7%) at the study clinics; 39 additional implants were removed at satellite clinics where removal details were not recorded.

## 4 Discussion

Our results demonstrated that Sino-implant (II) is a highly effective contraceptive method during the third, fourth and fifth years of use in this population of Chinese women. While we observed four pregnancies in Year 3 and 4, the overall rate was low (0.63 per 100 WY), with no pregnancies in Year 5. The low pregnancy rate over the three year period is consistent with the mean LNG concentrations remaining well above 200 pg/mL and at the level generally considered consistent with high contraceptive effectiveness [6]. However, the noticeable downward trend in the free LNG index (presumed to be more highly correlated with pregnancy prevention than total LNG) [7], [8], [9] suggests that the underlying risk of pregnancy may nonetheless increase somewhat over time. That said, great caution needs to be taken when interpreting PK trends from this study with its complex cohort design (i.e., different cohorts followed during the five-year time period) and noting the large inter-subject variability in LNG and SHBG measures.

In comparison to the trend toward decreasing pregnancy rates in the China cohort study presented here, the pivotal DR trial used for WHO PQ observed a significantly higher pregnancy rate in the fourth year (3.54 per 100 WY) than in the first 3 years combined (0.18 per 100 WY; p ≪.001). Although this difference between the two studies could have been due to simple random variation associated with a relatively rare outcome, a number of factors including age, weight, coital frequency, other covariates (e.g. unreported condom use for protection against STIs) or potential differences in genetic factors related to the PK and pharmacodynamics of LNG [10], [11], [12], may have led to true differences in pregnancy rates across populations. In a secondary analysis of the China data where we excluded follow-up data from 12 participants (2.4%) who reported condom use for STI protection or oral contraceptive use to control bleeding disturbances, pregnancy rates were virtually the same (data not shown).

Recruitment into the China cohort study, with its weaker design compared to the randomized controlled design we employed in the DR, may also have introduced selection bias. Women enrolled after using Sino-implant (II) for at least 2 years were potentially at different risk of pregnancy than a patient population assigned the product at enrollment into a traditional randomized contraceptive trial. However, prospective studies often suffer from similar selection biases, as a non-trivial percentage of women discontinue their contraceptive method well before reaching its intended duration of action due to side effects, desire for pregnancy, or other personal reasons. If the concern is related to more fecund implant users conceiving in the first 2 years and our study having enrolled women with lower fecundity, the trial in the DR has demonstrated an exceedingly low risk of pregnancy in the first 2 years; 0.00 per 100 WY (95% CI: 0.00–0.79) and 0.28 per 100 WY (95% CI: 0.01–1.55), respectively.

Another notable difference between the two studies was the incidence of implant breakage during implant removal even though we provided the same instructions across the two studies about pulling out implants without twisting. In the DR trial, Sino-implant (II) broke significantly more often than Jadelle (16.3% versus 3.1%; p ≪.001). In the China study, the Sino-implant (II) breakage rate (5.7%) was similar to the breakage rate in an earlier Chinese study (5%) [13], and comparable to breakage rates of contraceptive implants from other studies [14], [15].

Acceptability of Sino-implant (II) in terms of impact on menstrual bleeding is inconsistent across questions and difficult to interpret. While most participants (93.7%) reported their bleeding pattern to be acceptable during the study, over half who discontinued using implants during the study provided vaginal bleeding related reasons for discontinuation (bleeding disturbances (42.0%), decreased bleeding/amenorrhea (13.0%)).

WHO prequalification of Levoplant™ as a 3-year method was achieved in June 2017 based on the data we presented in this paper and the pivotal trial from the DR. Based on the significantly higher pregnancy rate during the fourth year of Sino-implant (II) use in the DR study, the WHO decision to prequalify the product for 3 years is appropriate. While the observational cohort design described here has some inherent weaknesses, we believe this approach has important supplementary value and as such, should be given regulatory consideration in future settings. For example, manufacturers could use this study design to extend labeled duration of use of existing approved products by enrolling women into a GCP compliant study as they approach their contraceptive method's labeled duration of use.
